# Beyond the game: social interaction as a bridge from sports to community cohesion and belonging

**DOI:** 10.3389/fspor.2026.1886326

**Published:** 2026-08-21

**Authors:** Zi Ye, Xiaoqiang Shi

**Affiliations:** Shanghai University of Sport, Shanghai, China

**Keywords:** active living, community belonging, community cohesion, psychosocial well-being, social interaction, sport participation

## Abstract

Although community sports are widely promoted to enhance social integration, the mechanisms linking individual participation to collective cohesion remain theoretically underdeveloped. Grounded in Social Capital Theory and Social Interaction Theory, this study seeks to explain how social interactions serve as a conduit for converting sports engagement into community assets. To achieve this, a cross-sectional design was adopted, utilizing validated measurement instruments to survey participants in community sports activities. Structural Equation Modeling was subsequently employed to uncover key mediating pathways. The empirical results demonstrate that social interaction acts as a critical mechanism linking sports participation to community outcomes. Specifically, both the frequency and quality of these interactions significantly influence community attachment and trust. These findings indicate that sustained interaction facilitates the accumulation of bonding and bridging social capital, thereby strengthening overall community cohesion and the sense of belonging. By clarifying these linkages, this research advances the understanding of community dynamics. The evidence compels policymakers to recognize community sports programs not merely as recreational activities but as a critical strategic intervention for achieving social cohesion goals.

## 1 Introduction

Community sports participation is widely recognized as a vital component of active living, yielding significant positive effects across physical, social, and psychological domains. Beyond the well-established benefits of physical fitness, community sports serve as a unique social environment where physical exertion intersects with complex interpersonal dynamics. Substantial evidence suggests that these settings can foster social integration and mitigate social inequalities by bringing together individuals from diverse backgrounds. Systematic reviews have consistently highlighted that the social nature of team sports, in particular, contributes to enhanced self-esteem and social interaction, positioning community sports as a potential catalyst for social well-being.

Despite these acknowledged benefits, the theoretical mechanisms linking individual participation to collective outcomes remain underdeveloped. A critical review of the literature reveals a disproportionate focus on mega-events, often overshadowing the nuanced dynamics within routine community settings. Furthermore, the conceptualization of social cohesion remains inconsistent across studies, with many frameworks failing to define the construct clearly or operationalize its measurement effectively. Consequently, there is a paucity of research examining the psychological dimensions of routine community sport participation, particularly regarding how these activities translate into community-level assets.

To address these gaps, this study grounds itself in Social Capital Theory and Social Interaction Theory. While previous qualitative research has touched upon the development of group belonging, the specific processes through which social interaction mediates the relationship between sports participation and community outcomes require further empirical validation. The current research aims to develop a theoretical-empirical framework that explains how the frequency and quality of social interactions within sports settings contribute to community cohesion and belonging. By clarifying these micro-macro linkages, this study seeks to provide a more robust foundation for understanding the social impact of community sports programs.

## 2 Literature review

### 2.1 Social capital theory and social interaction theory

Within the sport literature, social capital theory has become a dominant lens for explaining how sport generates social value. Sport settings—especially voluntary clubs and community programs—are widely characterized as sites in which networks, trust, and reciprocity can accumulate (1). Empirical work has linked sport involvement to social connectedness, social support, and broader civic participation (2), and systematic reviews of the psychological and social benefits of participation have informed conceptual models of “health through sport” in which social outcomes feature centrally (3). At the same time, scholars caution against romanticizing these effects: cohesion is not an automatic by-product of participation, and outcomes depend heavily on program design and on the quality of the relationships that sport cultivates (1, 2). For the present study, social capital theory frames community cohesion, sense of belonging, and community attachment as relational outcomes that sport participation may help to produce.

Whereas social capital theory specifies the relational resources at stake, social interaction theory addresses the interpersonal processes that produce them. Two strands are especially relevant. The first, symbolic interactionism, holds that people act toward others on the basis of meanings that arise—and are continually negotiated—through social interaction (4). In this view the self is not innate but emerges through interaction: individuals come to understand who they are by interpreting how others respond to them (5), and shared meanings and a common sense of belonging are constructed in and through everyday encounters (6). The second strand, social exchange theory, conceptualizes interaction as a reciprocal exchange of tangible and intangible benefits, in which repeated and balanced exchanges cultivate trust, obligation, and commitment over time (7). Notably, Blau (8) treated such exchange as a foundation of social cohesion, and exchange theory has well-recognized links to social capital research through its shared emphasis on networks, norms, and trust.

### 2.2 Residents' sport participation in community sports events

Sports participation, as a multidimensional concept, encompasses various forms of engagement and participation in sports activities. Existing literature has explored the essence of sports participation from different perspectives.

Nothnagle and Knoester (9) suggest that sports participation should be understood as a purposeful form of leisure, emphasizing its crucial role in fostering resilience during individual development. Moreover, the significant findings of this definition indicate that sports participation is not merely physical activity but also a key pathway for shaping personal character. Furthermore, the evidences suggest that Kay et al. (10) proposed a life cycle model of sports participation from the perspective of participation stages. The delineation of sports participation into discrete behavioral stages, including encompassing enrollment, maintenance, sustained engagement, transition, withdrawal, continued disengagement, and critical re-engagement. Framework shows participation patterns vary across life stages. However, the significant findings suggest that Teare and Taks (11) emphasize the comprehensive impact of sports participation on physical and mental health from the perspective of health promotion. In light of the evidence, sports participation have positive effects on physical, social, and psychological health, as well as academic performance, as a key form of leisure-time physical activity. Additionally, the results indicate that this perspective places sports participation within a broader health promotion framework. Notwithstanding these considerations, the significant evidence suggests that the multifaceted value of sports participation appears to extend well beyond purely physical outcomes. Evidence shows participation supports health promotion broadly.

From a sociological perspective, Puig (12) focuses on the social stratification characteristics of sports participation. Research on sports participation must consider the influence of sociodemographic and socioeconomic factors on participation patterns, as well as differences in sports participation among various social groups. This perspective reveals the social inequalities present in sports participation, providing a basis for developing more inclusive sports policies.

In this study, sports participation is defined as residents voluntarily choosing to participate in various community sports events. The focus is not on forcing residents to complete a specific amount of exercise or achieve a certain level of fitness, but rather on encouraging residents to actively participate in community events through innovative event content and diverse organizational forms. To measure respondents' sports participation, this study adapted the ACSM's Exercise Testing and Prescription (13). The sports participation questionnaire consists of three items: frequency, intensity, and time (14). Frequency, intensity and time serve not as physiological metrics, but as proxies for the degree of participation. This approach captures residents' actual behavioral involvement in community initiatives, reflecting how actively they are embedded in the community sport ecology (15).

### 2.3 Social interaction, community cohesion and community belonging

Within the context of community sport, social interaction acts as the fundamental psychological catalyst that transforms individual physical activity into profound collective experiences. Grounded in Social Identity Theory, shared sporting experiences enable individuals to derive a sense of belonging from group membership, where the formation of a collective identity fosters strong emotional bonds among participants (16, 17).

Social interaction in the field of sports is defined as an important mechanism for promoting the establishment of interpersonal relationships and the socialization process through sports participation (18). Yang et al. (19) applied social interaction theory to the analysis of behavioral patterns in urban running groups, found that the interaction process within the entire running group is accomplished through three aspects: self-reflection, adjustment with others, and a sense of group belonging as a core consciousness. Punch. et al. (20) examined the role of sports in promoting social capital from a cross-critical perspective, found that sports participation has a promotional effect on the development of social capital for immigrant women from culturally and racially marginalized groups, but this process is more challenging compared to local populations. Izquierdo and Anguera (21) conducted a mixed-methods study on interpersonal communication in sports, exploring the multimodal nature of interpersonal communication in sports and emphasizing the importance of interpersonal systems and social interaction in a sports context. Spaaij and Schaillée (22) draw on interactive ritual theory to find that the accumulation of emotional energy and bodily synchronization through face-to-face interaction can foster group cohesion, thereby opening the “black box” of sports-promoted development. However, they also note that this method remains limited in its explanation of class and race.

Community cohesion in sports research suggest that the ability to promote unity, cooperation, and collective identity among community members through sports activities demonstrate significant social value (23). Moreover, the findings from Räikkönen and Hedman (24) indicate that their novel conceptual framework appears to establish an important relationship between sports, shared spaces, community capacity, and community awareness. Furthermore, the evidence that sports serve as a unique social space indicates how individuals create and strengthen the key interactions and community relationships that appear to matter most. In light of these significant findings, the results indicate that this process will enhance the willingness and ability to collectively address challenges and seize opportunities while fulfilling important needs for belonging and purpose. Research shows community sports programs foster social cohesion. However, the evidence also indicate that such programs suggest a crucial role in providing opportunities for individuals from diverse backgrounds to engage in sports activities and significant social interactions. Thus, the findings demonstrate that these important programs appears to help break down social barriers and reduce social inequality by targeting underrepresented groups (25). Given that Rich et al. (26) have demonstrated key distinctions between different concepts of community in community-theorized research, the results appear to suggest that sports management studies and practice reveal important categories. Notwithstanding these results, the significant findings indicate that the evidence support community as a context, community as an outcome, community as a site of struggle or resistance, and community as a form of regulation or social control. Study shows framework supports understanding community cohesion in sports.

Community belonging in the field of sports is defined as the psychological experience individuals feel when participating in sports, where they perceive acceptance, value, and emotional connection with the group (27). In Spaaij (28) study, community sports served as a venue for refugee youth to negotiate a sense of belonging, fostering belonging across different levels—from sports teams and local communities to transnational contexts. Anderson-Butcher et al. (29) found in their theoretical study on how community sports programs improve the health of vulnerable groups that if community sports provide participants with opportunities to understand and connect with one another, participants will experience a sense of belonging and improved self-esteem, particularly for vulnerable or marginalized youth who have limited access and opportunities to these experiences. They feel noticed and understood by their peers and coaches. Sports are described as having the power to transform not only people 's lives but also communities; reaching where people live, improving those places, and fostering pride and a sense of belonging. Kyeremeh (30) noted that when people gather in shared sports spaces and build trust and common relationships, it also enables them to make more informed decisions about the challenges they face.

## 3 Hypotheses development

### 3.1 Sports participation and social interaction

Team sports participation consistently improves social skills, peer relationships, and community engagement compared to individual activities, with strongest benefits during adolescence (31). For the adult group, Yang et al. (19) conducted interviews and thematic analysis with members of a marathon running group, found that running communities promote a sense of belonging and internalization through standardized organizational structures, unique ritual cultures, and emotional atmospheres that shape group behavior, thereby promoting sustained social interaction. In repetitive participation, Theodorakis et al. (32) believed that people invest more time and resources in repetitive sports events and are more likely to increase social interaction. For individuals with depression, participation in team sports during youth is associated with improved social skills, better peer relationships, and reduced depressive symptoms compared to individual sports (33). Therefore, participation in sports continues to improve social connections. Accordingly, we propose the following hypothesis:

H1: Among community sport event participants, sports participation positively influences social interaction.

### 3.2 Sports participation and community cohesion

Research consistently demonstrates that sport participation serves as a significant catalyst for enhancing community cohesion through multiple interconnected mechanisms (23). Community sports programs have been found to significantly enhance social capital, which in turn promotes social cohesion, with participants reporting increased trust, improved social networks, and a stronger sense of community (34). Jaramillo et al. (35) analyzed the Facebook social network dynamics of the Recreovía sports program and found that for every additional participant (user), an average of 1.73 new online friendships were formed. This indicates that residents' participation in sports significantly promotes social cohesion through the rapid expansion and strengthening of social connections. In addition, based on a systematic review of multiple studies, the research found that when sports programs are conducted in a culturally appropriate community development format, they significantly enhance social cohesion among residents by cultivating local knowledge and skills, creating dialogue mechanisms, and strengthening social bonds (36). Another study surveyed European sports programs and mentioned that if sports practices focus only on social skills at the individual level, then systematic construction of community cohesion requires attention to the arrangement of sports programs and the implementation of sports policies (37). Accordingly, we propose the following hypothesis:

H2: Among community sport event participants, sports participation positively influences community cohesion.

### 3.3 Sports participation and community belonging

Sport participation creates substantial opportunities for developing community belonging through shared experiences and social identity formation (24). Yun et al. (38) conducted in-depth interviews with young people with disabilities and their parents and found that wheelchair sports enable young people with disabilities to gain a deep sense of belonging through three mechanisms: creating inclusive communities, reshaping participants' self-perceptions, and fostering identity recognition. Eather et al. (39) conducted a systematic review and found that adults who participate in elite-level team sports in their communities significantly improve their sense of belonging through enhanced social interaction, and team sports are superior to individual sports in terms of psychological and social benefits. Persson and Eriksen (40) believed that participating in sports as part of daily life strengthens a sense of belonging. Mansell et al. (41) suggested that incorporating anti-racism as a guiding value in community sports and recreation clubs will promote healing, decolonization, and equity in Indigenous communities, as well as a sense of community belonging. In addition, post-war divided communities significantly reduced team hostility through inter-ethnic mixed team sports events combined with trust-building, and catalyzed a sense of belonging across communities through cooperative competition (42). Therefore, we propose the following hypothesis:

H3: Among community sport event participants, sports participation positively influences community belonging.

### 3.4 Social interaction and community cohesion

Social capital theory provides an important theoretical foundation for understanding the relationship between social interaction and community cohesion in sports. Moustakas (43) integrated social capital theory, social identity theory, and ecosystem theory through research on how European sports promote social cohesion to explain how community sports programs enhance social cohesion, emphasizing the importance of social networks, group identity, and environmental interaction. The study found that organizations adopt a perspective on social cohesion, primarily focusing on social relationships, tolerance, and mutual assistance. Sánchez-Santos et al. (44) suggested that the bidirectional relationship between sports participation and social capital building will demonstrate important patterns. Moreover, the significant findings indicate that high-frequency sports participation (three or more times per week) appears to show increased social connections and the likelihood of forming close friendships. Furthermore, the evidence above suggests that organized sports within formal organizations demonstrate more effective social capital building than independent participation. In light of these results, Spaaij and Schulenkorf (45) indicated that sports development practices conducted in a conscious, culturally relevant manner effectively promote increased social cohesion. Study shows micro-processes reveal social capital development. Tacon (46) suggested that the key findings from a case study of three voluntary sports clubs in the UK have demonstrated the significant micro-processes of social capital development. Given that the evidence demonstrates 15 months of observation work at a cricket club, a football club, and a tennis club, the results indicate that in-depth interviews with club members would support the primary mechanisms outlined by Portes. However, the significant findings indicated that formal club structures provide a stable framework for social interaction. Nevertheless, the key evidence indicates that true social cohesion demonstrate development through informal interactions and shared experiences (47). Results show formal structures support cohesion. Therefore, we propose the following hypothesis:

H4: Among community sport event participants, social interaction positively influences community cohesion.

### 3.5 Social interaction and community belonging

There is a reciprocal relationship between social interaction and community belonging. Eime et al. (3) conducted a systematic review and found that the most common psychological and social health benefits of sports participation include improved self-esteem, social interaction, and reduced symptoms of depression. Warner and Dixon (48) conducted a grounded theory analysis of college athletes in the United States and found that competitive and social spaces can strengthen mutual interaction among members, which is the core driving mechanism of belonging. Even in virtual sports communities, by reinforcing collective identity and optimizing interaction interfaces, the frequent digital interactions among fans in virtual sports fan communities can be transformed into a sense of belonging to the digital community (49). What's more, It is important to note that expanding the scope of sports clubs and having community organizations host events is crucial. Skinner et al. (50) argued that under neoliberalism, governments create structured interaction spaces for disadvantaged groups through institutional design, enabling cross-class collaborative movements to become social capital and catalyzing a sense of belonging based on shared space and collaborative identity. Therefore, we propose the following hypothesis:

H5: Among community sport event participants, social interaction positively influences community belonging.

### 3.6 The mediating role of social interaction

Participation in sports can foster meaningful interpersonal relationships, thereby enhancing participants' psychological attachment to their broader community environment (51). Social interaction in sports activities enables individuals to develop relationships, share experiences, and establish social connections that extend beyond the immediate sports environment, ultimately cultivating a deeper sense of community belonging and community cohesion. Marlier et al. (52) found that social interaction positively mediates the relationship between sports participation frequency and community attachment by enhancing social capital development. Similarly, Schulenkorf (53) noted that the quality of social interaction during sports participation significantly influences community connections by establishing trust and mutual support networks. Team sports activities generate higher levels of social interaction than individual sports activities, which in turn enhances participants' community identity and civic engagement. However, existing quantitative research on community sports event participation is limited, and most existing studies treat social interaction as a concomitant phenomenon of sports participation rather than a specific quantifiable mediating variable. Therefore, we propose that social interaction is the key mechanism through which sports participation translates into enhanced community identity, as it promotes the establishment of social bonds and the development of shared identity, thereby strengthening community attachment. Based on studies above, we propose the following hypothesis:

H6a: Among community sport event participants, social interaction mediates sport participation and community cohesion.

H6b: Among community sport event participants，social interaction mediates sport participation and community belonging.

**Figure 1:**
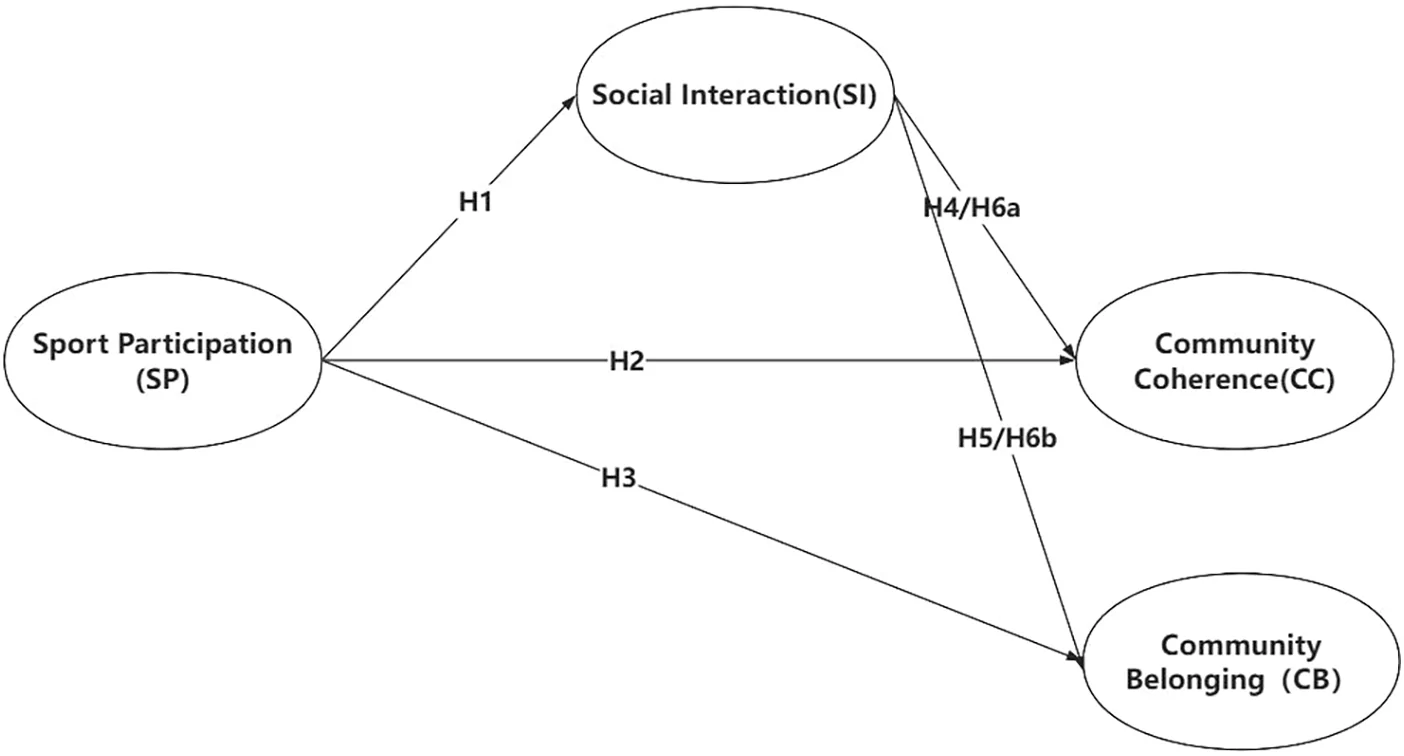
Research model.

All of the above hypothetical research models are shown in Figure 1

## 4 Method

### 4.1 Measurement

The questionnaire includes four latent variables with 13 items, which are shown in Appendix A, and some demographic characteristics (e.g., gender, age, educational background, monthly income, and sport event frequency). The survey covers the validated measurements of sport participation, social interaction and community cohesion. These reflective measurement items are assessed using a 5-point Likert scale (1 = “strongly disagree,” 5 = “strongly agree”). To measure respondents' sports participation, we adapted the ACSM's Exercise Testing and Prescription guidelines. The sports participation questionnaire comprises three items: frequency, intensity, and duration. The scale combined different scales to create a community sports event social capital scale, including social interaction (54, 55), community cohesion (56, 57), and community belonging (58), For the community-based sports social capital measurements, three reflective measurement items were used for all three scales.

### 4.2 Data collection

This study was approved by the ethics committee of the institution where the primary author is affiliated (Approval No. 102772025RT196). Written informed consent was obtained from all participants, and all data were processed anonymously to protect privacy. The questionnaire was originally written in English, then translated into Chinese and independently back-translated to ensure linguistic accuracy and cultural appropriateness. To ensure the accuracy and practicality of the questionnaire, all measurement items were derived from previous scales and appropriately modified based on the context of community sports events. The study suggested that data collection in China required the back-translation method proposed by Brislin (59). However, the process indicated that one translator rendered the English items into Chinese, while another translator converted them back into English. Furthermore, the significant findings from comparing the two versions demonstrate that no meaningful differences in meaning were present. In light of this verification process, the primary research questionnaire indicates that trained staff distributed it following approval from the event organizing committee. Distribution shows questionnaire reached participants July–August 2025. Moreover, the results suggest that participants were recruited through on-site intercept sampling across various venues. Given that the evidence demonstrates direct community involvement, the key findings indicate that survey participants were residents directly engaged in the community sports event. Thus, the significant data suggests that this approach appears to support direct relevance to the research objectives. Sampling method ensures participant relevance to study goals.

## 5 Results and analysis

### 5.1 Descriptive statistics

The demographic information of respondents is summarized in Table 1. Of the 478 valid survey samples, 287 (60.04%) were female. In terms of age, all age groups are distributed across the sample, with the largest proportion being the 30–45 age group at 44.98%, followed by the 18–30 age group at 42.47%. The port event participants were the young and middle-aged group under 45 years old (87.45%). More than 85% of the respondents held a bachelor's degree or above. The monthly income was mainly in the group of CNY 3,001–7,000 (56.06% in total). Over half of the respondents said that they participate in sport events at least once every two weeks, with 32.64% participating once every two weeks and 25.73% participating once a week, both of which are mainstream frequencies for sport participation. In summary, the participants engaged in community sports events show the characteristics of younger, well-educated, and higher income demographic groups, which is consistent with the demographic characteristics of the sample align with those reported by the organizing committee (Shanghai Administration of Sports, 2024). Therefore, the participants in this study are broadly consistent with organizer-reported demographics.

**Table 1:** Demographics of respondents (*N* = 478).

Type	Item	Frequency	Percentage
Gender	Male	191	39.96%
	Female	287	60.04%
Age	Below 18	12	2.51%
	18–30	203	42.47%
	30–45	215	44.98%
	46–60	45	9.41%
	Over 60	3	0.63%
Education Background	Second school or below	15	3.14%
	High school	56	11.72%
	Bachelor	314	65.69%
	Master and PhD	93	19.46%
Monthly Income (CNY)	Under 3,000	71	14.85%
	3,001–5,000	135	28.24%
	5,001–7,000	133	27.82%
	7,001–9,000	69	14.44%
	Over 9,000	70	14.64%
Sport Event Frequency	Once a week	123	25.73%
	Once every two weeks	156	32.64%
	Once a month	125	26.15%
	Occasionally	62	12.97%
	Never	12	2.51%

### 5.2 Measurement model

First, the results of the indicator weight assessment for the measurement model were satisfactory. Second, the VIF values for all measurement items were less than 3, indicating no significant multicollinearity issues among the measurement items. Therefore, the reliability and validity of this measurement model are good. Third, the reliability and validity assessment results of the reflective measurement model (Tables 2, 3) show that the Cronbach's *α* and Composite Reliability (CR) values for variables such as “sport participation” are all greater than 0.7, indicating that the measurement models have good internal consistency reliability. All factor loadings of the observed items are greater than 0.7, and all AVE values of the latent constructs are greater than 0.5, indicating that the measurement models have good convergent validity. In terms of discriminant validity, the square roots of all latent construct AVE values are greater than the correlation coefficients between each latent construct and any other latent construct, indicating that the measurement models have good discriminant validity.

**Table 2:** Reliability and convergent validity.

Variable	Item	Factor Loading	CR	AVE	Cronbach's *α*
Sport Participation (SP)	SP1	0.875	0.883	0.808	0.881
	SP2	0.923			
	SP3	0.898			
Social Interaction (SI)	SI1	0.86	0.82	0.734	0.819
	SI2	0.837			
	SI3	0.873			
Community Cohesion (CC)	CC1	0.866	0.815	0.731	0.815
	CC2	0.812			
	CC3	0.884			
Community Belonging (CB)	CB1	0.857	0.836	0.668	0.834
	CB2	0.819			
	CB3	0.797			
	CB4	0.794			

**Table 3:** Reliability and convergent validity.

Construct	SP	SI	CC	CB
SP	0.899			
SI	0.362**	0.857		
CC	0.348**	0.581**	0.855	
CB	0.357**	0.43**	0.455**	0.817

The diagonal elements are the square roots of the AVE values for each latent construct, and the remaining elements are the correlation coefficients between latent constructs. **P < 0.01.

### 5.3 Structural model

This study employs the partial least squares (PLS) algorithm and utilizes the SmartPLS 4.1.0.6 data analysis software to test the significance levels of path coefficients (Table 4). The results indicate that participation in sports has a significant positive impact on social interaction (*β* = 0.362, *P* < 0.001), suggesting that engaging in sports can effectively promote individuals' social interaction behaviors. Social interaction, as a mediating variable, has a significant positive impact on community cohesion (*β* = 0.523, *P* < 0.001) and community belonging (*β* = 0.346, *P* < 0.001), indicating that social interaction can effectively enhance community cohesion and belonging among community members.

**Table 4:** Path coefficient and its significance.

Path	*β*	SD	T	LLCI	ULCI	*P*
PC → CC	0.159	0.042	3.774	0.076	0.242	**
PC → CCB	0.232	0.041	5.604	0.147	0.311	**
PC → SI	0.362	0.05	7.183	0.259	0.459	***
SI → CC	0.523	0.054	9.626	0.412	0.623	***
SI → CCB	0.346	0.053	6.545	0.241	0.445	***
PC → SI → CC	0.189	0.038	4.972	0.118	0.266	**
PC → SI → CCB	0.125	0.031	4.048	0.07	0.189	**

** *P* < 0.01.

*** *P* < 0.001.

Additionally, sports participation also exhibited a direct positive relationship with community cohesion (*β* = 0.159, *P* < 0.01) and community belonging (*β* = 0.232, *P* < 0.001). From the confidence interval analysis, the 95% confidence intervals for all path coefficients, both the bias-corrected and percentile-based, did not include zero, further validating the robustness of the relationships between paths.

Furthermore, the mediating roles of social interaction were confirmed. Specifically, the indirect effect of sports participation on community cohesion via social interaction was significant (*β* = 0.189, *P* < 0.01), as was the indirect effect on community belonging (*β* = 0.125, *P* < 0.01). From the confidence interval analysis, the 95% confidence intervals for all path coefficients did not include zero, further validating the robustness of the relationships between paths.

**Figure 2:**
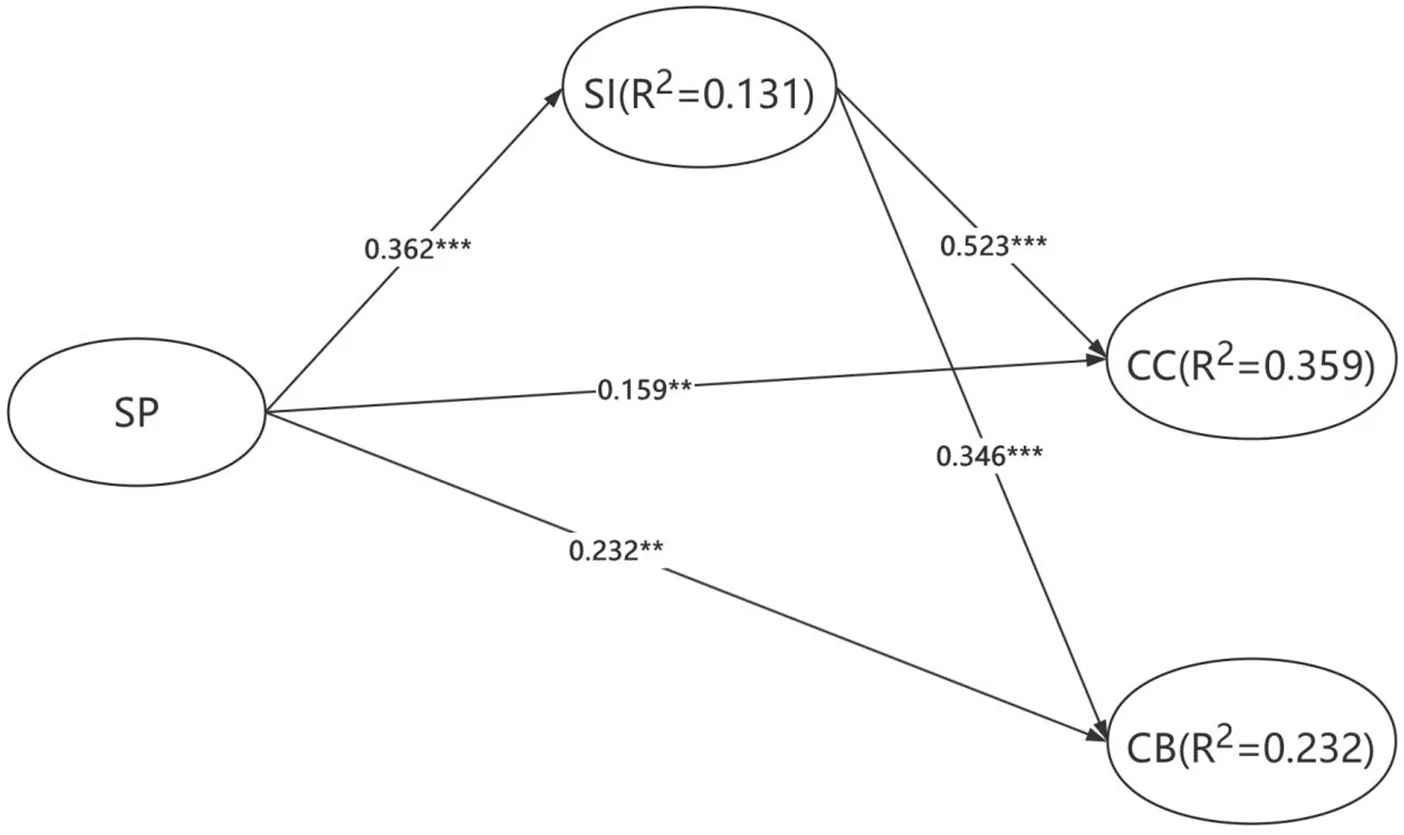
Results of the research model. *** *P* < 0.001, ** *P* < 0.01. The mediation effect (H6) is shown in italics, and within the brackets.

In terms of model explanatory power, the R^2^ value for social interaction was 0.131, for community cohesion reached 0.357, for community belonging was 0.232, indicating that the model has strong explanatory power for community cohesion. The predictive relevance of the model was further supported by Q² predict values of 0.125 (social interaction), 0.117 (community cohesion), and 0.121 (community belonging), all exceeding the threshold of zero. Additionally, the model fit was satisfactory, with a standardized root mean square residual (SRMR) of 0.058. Comprehensive analysis indicates that sports participation can effectively enhance community cohesion and belonging levels through the mediating mechanism of promoting social interaction (Figure 2).

## 6 Discussion and implications

### 6.1 Discussion of findings

This study proposes a comprehensive model explaining how sports participation contributes to social development outcomes, and it is theoretically important. The key finding is that the proposed theoretical framework receives empirical support, establishing a clear link between individual participation in sports and broader psychosocial benefits. Evidence shows that participation in sports acts as a key mechanism for strengthening social bonds, fostering community interaction, and enhancing social cohesion. The findings further indicate that sports help fulfill individuals' need for connection and transform personal relationships into a collective spirit of community cohesion.

However, the model reveals that sports participation influences social outcomes through both direct and indirect pathways. In light of the data supporting social capital theory, Putnam (60) stated, “In this context, the outcomes that participation in sports creates can point to social resources that extend beyond the immediate sports environment.” Similarly, this study finds that the relationship between sports participation and social capital yields meaningful social resources beyond the direct sporting context. Organized sports provide a structured avenue for social connection. Beyond these results, the evidence confirms that sports function as a powerful system for participation, utilizing the strong emotions and physical integration generated during sports activities to foster meaningful social connections and psychological bonding. Therefore, the intensity of these emotions appears closely tied to social bonding outcomes.

The mediating role of social participation further clarifies how sports activities shape social outcomes. Furthermore, the results indicate that these outcomes are consistent with Coleman (61) theory of social capital; This theory emphasizes the importance of social and personal relationships in promoting the well-being of all people. However, the significant result indicates that mere participation in games is not sufficient to improve social outcomes. The quality and strength of social relationships foster a sense of unity and belonging. While the meta-analysis suggests that social components are good predictors, the evidence aligns with the recent research by Karstensen et al. (15) concluded that social components of sports participation are better predictors of social outcomes than the frequency of participation. This emphasis on the qualitative aspect of social ties resonates with the development of measurement tools by Forsell et al. (62), who argued that capturing the nuances of social capital in recreational settings is essential for understanding community dynamics.

Overall, the results support the model 's validity. Furthermore, the significant variation explained by the internal factors indicate the robustness of the proposed learning model, and the results suggest that participation in sports is an important factor in community development programs. These findings are consistent with earlier work by Perks (63) and Tonts (2), who identified a positive relationship between participation and social outcomes but did not examine the underlying processes. By illuminating these processes, this study reveals how sports participation connects social change, community resilience, and individuals' perceptions of their place within sports communities. Yet, it is crucial to acknowledge the duality of these processes. As demonstrated by Burns et al. (64) in their study of Western Australian clubs, strong “club connection” among young adults can simultaneously foster belonging and normalize excessive alcohol consumption. This suggests that not all forms of social cohesion are inherently beneficial. Base on these findings, future research should move beyond establishing correlations and begin examining the conditions under which sports participation generates inclusive and sustainable social outcomes.

### 6.2 Theoretical and practical implications

This study suggest that its findings make a significant theoretical contribution to community development and the sociology of sport, while also providing important practical insights for policymakers and professionals in the field of community sport.

Moreover, the significant results indicate that two distinct theoretical contributions emerge from this work. Furthermore, the evidence demonstrate that the first contribution expands the theoretical framework of community sport research. In these findings, the model suggests that it primarily integrates and extends social capital theory and self-perception theory. Study shows participation links to outcomes. However, the significant findings indicate that by empirically validating the link between community cohesion and community belonging, this study demonstrates that organized physical activity serves as a catalyst for the development of bonding and bridging social capital. This function of sports as a tool for social inclusion and public health aligns with the editorial perspective by Corvino et al. (65), who advocate for sport as a multifaceted instrument for societal well-being. Additionally, the evidence suggest that sport serves as a venue for repeated social interactions that strengthen community networks and foster collective identity. Thus, the results demonstrate that this principle aligns with social identity theory, though it has been applied only to a limited extent in the context of public sports. Given that the evidence indicates social interaction plays a mediating role, the significant findings suggest that social interaction is identified as a key mediator, as indicated by hypotheses H4/H6a and H5/H6b. Evidence shows mediation explains outcomes. Nevertheless, the results indicate that this mediating mechanism explains how participation in sports leads to better community outcomes. Therefore, the significant findings demonstrate that it is not simply the act of participating in sports, but rather the social processes generated by these activities, that matter most.

Moreover, the evidence suggest that mutual trust networks support community cohesion and a sense of belonging. In light of these key results, the study indicates that this finding extends understanding beyond direct-effect models and aligns with contemporary socio-ecological frameworks that emphasize process-based mechanisms in health and community development. Research shows model supports practice. Furthermore, the validated model suggest that it offers a structured framework for translating the results into practice and strengthening community cohesion. However, the significant findings demonstrate that when planning programs, organizers need to develop activities that foster meaningful social interactions. Given that the evidence supports deeper engagement, the results indicate that programs must go beyond mere physical exercise and create regular opportunities for repeated, deep engagement. Thus, the important findings suggest that it is essential to train organizers and instructors to focus more on fostering positive social interactions and strengthening the psychological bonds among participants than on technical instruction. This is particularly pertinent for rural settings; as Mountifield (66) notes, elevating the capacity of local clubs to manage these social dynamics is vital for policy considerations aimed at enhancing social cohesion. Moreover, the key findings suggest that social, economic, and cultural barriers must be removed to achieve this goal. Therefore, the evidence demonstrates that by integrating these approaches, policymakers can develop inclusive sports and social programs that leverage organized sports as vital communications infrastructure to strengthen community cohesion.

### 6.3 Limitations and future directions

While this study provides valuable insights into the relationship between physical activity, social interaction, and community building, it is also important to acknowledge its limitations. However, these limitations also pave the way for higher-quality research in the future.

First, the findings of this study are based on a small sample of individuals from a single cultural and geographical context. The strength of the hypothesized pathways (e.g., H1, H4/H6a, and H5/H6b) vary according to cultural and socioeconomic circumstances, as well as the type of community. In future research, more diverse, international, and representative samples of individuals should be used to test the validity of this conceptual model, so that the findings are more generalizable. Future studies should also consider the distinction between urban and rural contexts, as Eather et al. (39) found significant variations in health-related quality of life and the contribution of sports across different Australian locales.

Second, although a case study is a good way to validate a prototype, it leaves much room for drawing broad conclusions about its underlying processes. This theoretical model suggests that participation in games also creates social interaction, which in turn strengthens community cohesion and empathy. However, reverse causality also be present. (For example, individuals with a strong sense of community is more likely to participate in local games). Longitudinal studies and experimental interventions are also needed to empirically validate the proposed hypotheses (H1–H6b). For example, future research could monitor changes in the population over time following the launch of new gaming applications.

Third, this study is a significant milestone in human-computer interaction. Although this study shows that social interaction is an important indicator, future research should investigate people's perceptions more deeply to examine other variables that affect their participation in social games. Furthermore, researchers should investigate how social recognition works to understand how relationships with game teams enhance players' effort and sense of unity. It is useful to recognize perceived warmth and satisfaction with participation as two key indicators in the study. This helps to clarify the importance of both personal relationships and intrinsic satisfaction. Furthermore, future research should extend beyond the participants to also monitor the reflections of teachers and organizers, who are identified as relevant contextual factors. To explore how participation in games can promote social relationships and unity, and to investigate how a collaborative environment for creative work enhances satisfaction, participation, and unity.

## Data Availability

The raw data supporting the conclusions of this article will be made available by the authors, without undue reservation.

## Appendix

**Table A:** Questionnaire items and sources.

Dimension	Item	Source
Sport Participation (SP)	(SP1) I frequently participate in local community sports events.	Gabriel et al. (14)
	(SP2) The local community sports events I participate in involve high-intensity exercise.	
	(SP3) The local community sports events I participate in require significant time.	
Social Interaction (SI)	(SI1) I maintain close relationships with other community members.	Narayan and Cassidy (54); Onyx and Bullen (55)
	(SI2) I can quickly identify community members with whom I frequently interact.	
	(SI3) I have frequent interactions with non-family members within the community.	
Community Cohesion (CC)	(CC1) Residents in my community are willing to help each other.	De Silva (57); Lochner et al. (56)
	(CC2) Residents in my community are highly enthusiastic about participating in community affairs.	
	(CC3) Relationships among residents in my community are harmonious.	
Community Attachment (CA)	(CA1) Residents in my community are interested in events occurring within the neighborhood.	Grootaert and Van (58)
	(CA2) Residents in my community feel a sense of belonging to the neighborhood.	
	(CA3) Residents in my community enjoy living in the neighborhood.	
	(CA4) Residents in my community feel proud to tell others where they live.	
